# Endoscopic Surgery for Delayed Wound Healing After Achilles Tendon Suture Repair: A Report of Three Cases

**DOI:** 10.1111/os.12995

**Published:** 2021-04-07

**Authors:** Yu‐ping Yang, Hong‐yu Chen, Guo‐qing Cui, Xiao‐lei Xiu, Xiao‐peng Liu, Hua An, Ying‐fang Ao

**Affiliations:** ^1^ Department of Sports Medicine Peking University Third Hospital, Institute of Sports Medicine of Peking University, Beijing Key Laboratory of Sports Injuries Beijing China; ^2^ Department of Sports Medicine Peking University Third Hospital‐Chongli Zhangjiakou city, Hebei province 076350 China; ^3^ School of Clinical Medicine Peking University of Medical Department Beijing China; ^4^ Department of Hand Surgery Cangzhou Integrated Traditional Chinese and Western Medicine Hospital Cangzhou China

**Keywords:** Achilles tendon suture repair, Delayed wound healing, Endoscopy

## Abstract

**Background:**

Delayed wound healing is a potention complication after Achilles tendon suture repair and occurs for various reasons. The conventional treatment for delayed wound healing is open surgery, but patients face long recovery times and postoperative care is difficult.

**Case Presentation:**

This report presents three patients who were seen at our institute from April 2008 to October 2017 due to long‐term non‐healing wounds after surgery. All three patients had undergone surgery at least 2 months previously. We performed endoscopic surgery on these patients. After the operation, patients received less antibiotics and simpler care than would be required for conventional open surgery. There is no need to keep the wound open after the operation or perform wound cleaning for multiple times at the same time, which can reduce healing time. At the last follow up, all postoperative scores among the patients were significantly improved compared to before surgery. The Achilles tendon total rupture scores were excellent and the American Orthopedic Foot and Ankle Society scores were satisfactory, indicating improvements in Achilles tendon function and movement in patients after surgery.

**Conclusion:**

Our case reports demonstrate that arthroscopic treatment for delayed wound healing after Achilles tendon suture repair is satisfactory and reliable; frequent opening of the wound for cleaning is not required after the operation, thus reducing the healing time.

## Graphical abstract

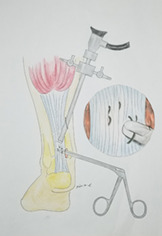

Delayed wound healing or poor healing is a potential complication after Achilles tendon suture repair. Non‐surgical methods are suitable for patients with mild symptoms, such as the use of sulfadiazine silver cream and negative pressure wound treatment. However, for patients who cannot be cured with non‐surgical treatments, the conventional treatment is open surgery. Debridement is used in surgery, and the wound is kept open for routine irrigation and dressing after the surgery until the tissue defect is repaired several days later. We pioneered the use of arthroscopy to treat patients with delayed wound healing after Achilles tendon suture repair. This article reports on three patients with delayed Achilles tendon wound healing who were treated with arthroscopy in our institution. We summarize our experience in treating the abovementioned patients and evaluate the advantages of arthroscopic treatment.

## Introduction

The Achilles tendon is one of the most vulnerable tendons because of the poor blood supply to the middle and lower segments^1^, ^2^, ^3^. Acute Achilles tendon ruptures are most commonly treated surgically, mainly because nonsurgical treatment has been demonstrated to be associated with greater risk of rerupture^4^. However, the rate of complications after Achilles tendon suture repair surgery is high, at approximately 28.47%; complications include foreign body reaction, skin necrosis, scar adhesion, skin laceration, and delayed healing as a result of reinjury^5^, ^6^. The wound healing usually takes several months through conventional surgical dressing methods, thus increasing the possibility of infection^7^. When open debridement is used, healing by first intention is unlikely because of the contaminated incision; a large wound is created, so that healing by first intention cannot occur and patients face long recovery times^8^.

When they undergo conventional treatments, patients take longer to recover and postoperative care is more difficult, indicating a need for the development of new techniques to safely and effectively treat delayed wound healing. Therefore, in 2008, we began performing arthroscopic debridement at our institute for delayed wound healing due to skin necrosis. The whole process, including the diagnosis and treatment, was explained to the three patients, and they provided informed consent before surgery and agreed to the postoperative follow up. Our endoscopic technique not only has a better surgical outcome but also greatly reduces the difficulty of postoperative care. The purpose of this study was to analyze the clinical efficacy of the method, to serve as a reference for clinical and surgical decision‐making and prognosis assessment.

## Case Presentation

### Case 1

The present patient was a 60‐year‐old Chinese man who had ruptured his Achillies tendon 4 months prior and underwent an incision and reconstruction in our institution in 2008. Before the rupture, local hormone injections had been used to treat Achilles tendon periarthritis. Considering that the rupture site was only 1.5 cm away from the posterior superior process of the calcaneus, the defect was large, approximately 4 cm, and the remaining scar tissue was fragile, so the gastrocnemius aponeurotic flap was used to rebuild the Achilles tendon. During the operation, several no. 1 and no. 4 silk sutures were used to fix the aponeurotic flap and eliminate the gap between it and the remaining scar tissue at the front.

When the sutures on the wound were removed 2 weeks after the operation, it was found that the skin was damaged in two places and purulent secretions were exuding. Because this was a local infection, no bacterial culture of secretions was performed. Neither defect was in the incision, and the proximal defect was far from the surgical incision. This incision was found to have ischemia and blackening during dressing changes. After 3 months of local dressing changes, there was no obvious improvement, so endoscopic treatment was performed.

**Fig. 1:**
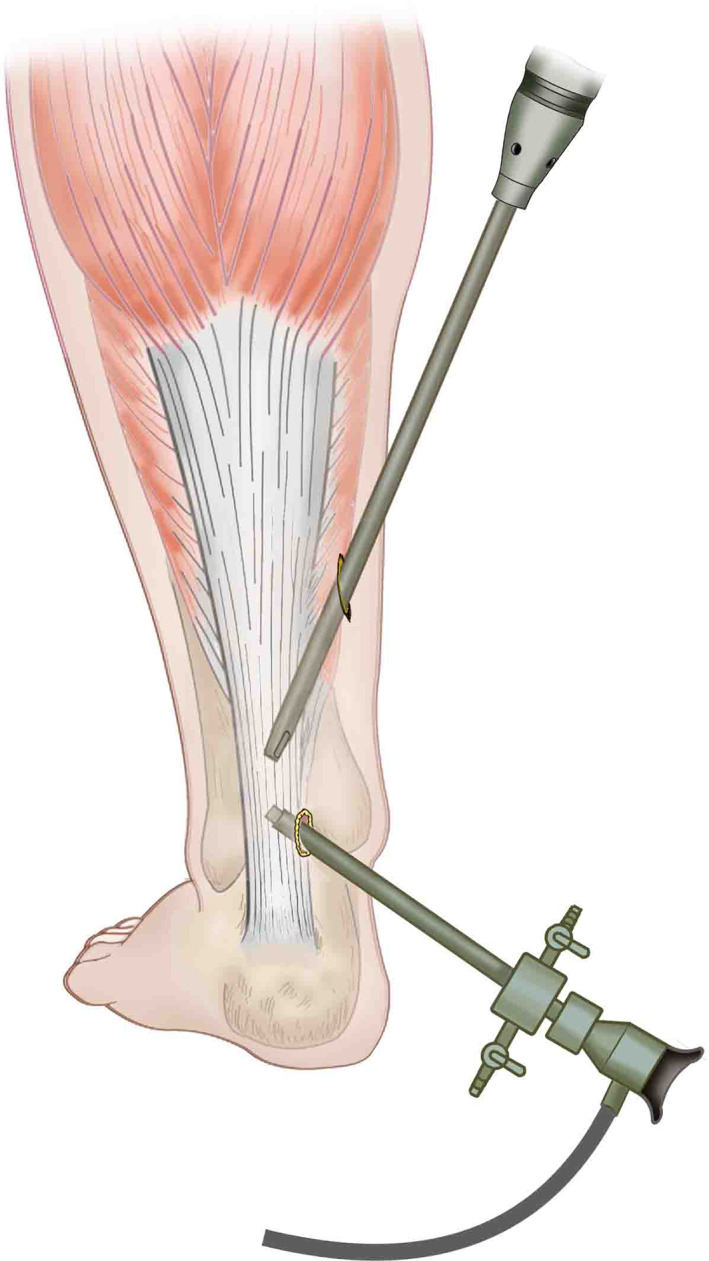
Arthroscopic approach.

**Fig. 2:**
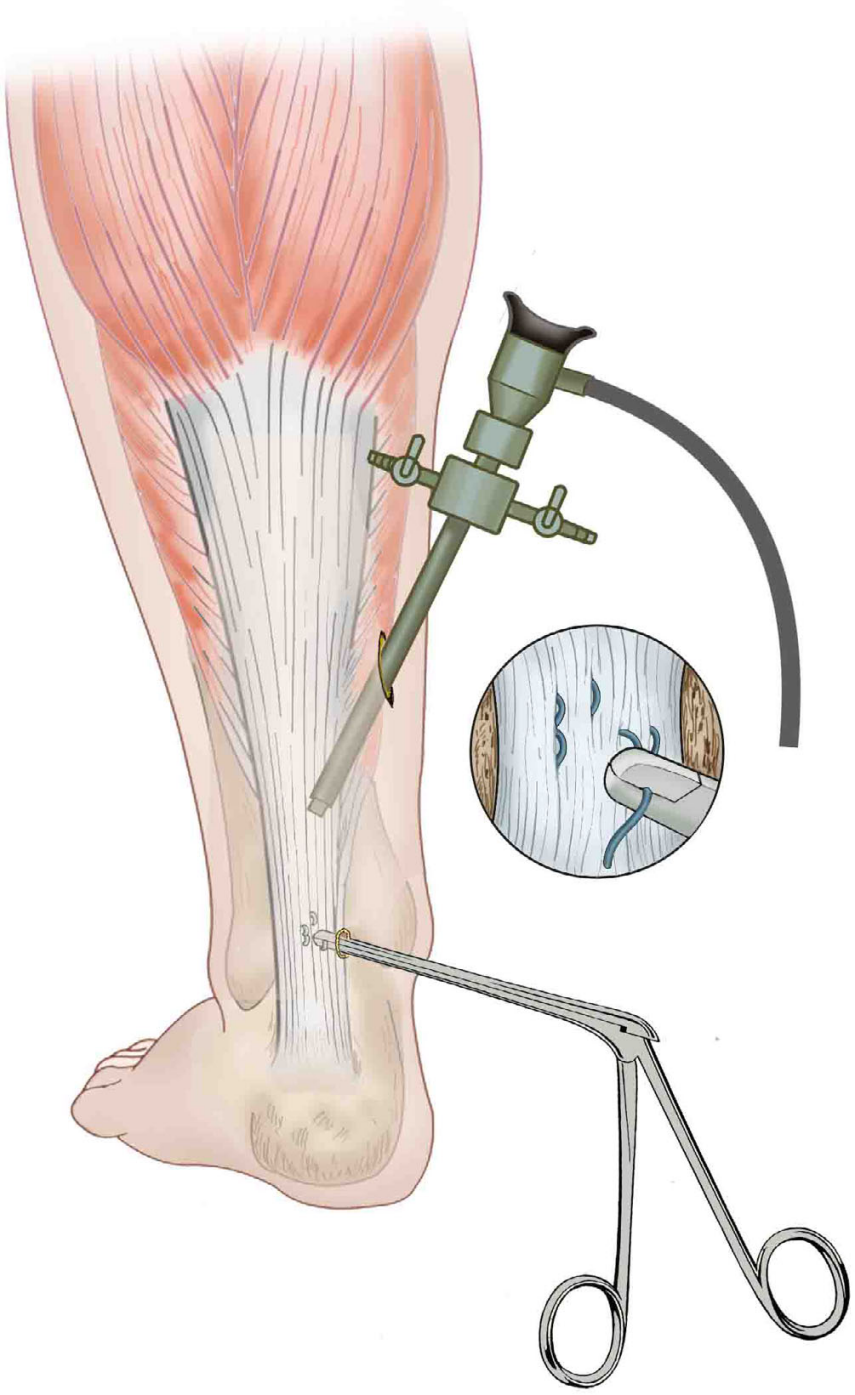
Schematic of treatment during arthroscopy.

The patient was placed in a prone position and received spinal anesthesia. A pneumatic tourniquet was applied to the affected lower extremity. The patient already had a defect into which the surgical instrument could be inserted. The other port selected was located at the top of the other side of the original defect (Fig. 1). Next, the inflammatory tissue was cleaned. Recent preoperative MRI images were used to identify areas with Achilles tendon weakness and degeneration. After the lesion was identified, the damaged tissue was cleaned by curettage, during which time the tendon was protected. All nonabsorbable sutures in the surgical field were removed (Fig. 2). After the wound and Achilles tendon tissue were rinsed, primary suturing of the wound was performed. A rubber strip was placed in the proximal wound and removed 1 day after the operation.

We used intravenous second‐generation cephalosporin as the prophylactic antibiotic for 1 week and then switched to oral medicine for 2 weeks; a short leg plaster was applied for 2 weeks. The wound suture was removed 3 weeks after the operation. The patient performed ankle joint range‐of‐motion exercises without weight‐bearing for 2 weeks after that and then began to walk with weight‐bearing while using a heel cushion. The cushion heel thickness was 2 cm from weeks 5 to 8 after the surgery, and the patient used crutches; from weeks 9 to 12 after the surgery, a cushion heel thickness of 1 cm was used. The patient stopped using crutches 3 months after the surgery. To minimize the risk of falling, the patient was instructed not to jump or lift the heel for the next 3 months. After that, he began to walk normally, and 6 months after surgery, he could do double heel exercises. He started doing single heel exercises 12 months after surgery.

The patient's recovery was satisfactory at the last the follow up in 2011 (3 years after the operation). The wound had healed completely, and no abnormality was noted during heel lifting or other movements.

### Case 2

**Fig. 3:**
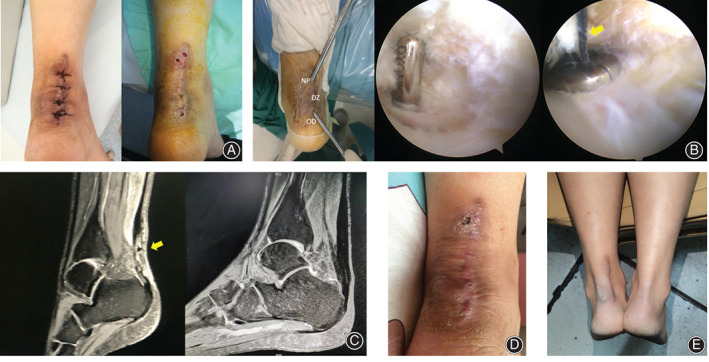
(A) The situation after Achilles tendon suture repair and the wound situation after one and a half months. (B) Intraoperative approach and arthroscopic findings. The yellow arrow indicates that the thread that caused delayed wound healing is being removed. (DZ, delayed wound healing zone; NP, new portal; OD, original defect.) (C) Preoperative and postoperative (6 months later) Achilles tendon MRI. The arrow indicates a high‐signal area, which, considering the patient's delayed wound healing and inflammation, may represent foreign body response caused by a suture. (D) The wound 1 week after surgery. (E) The current wound condition (4.5 years after surgery) which has completely healed.

The present patient was a 39‐year‐old Chinese man. After the patient started to get out of bed 1.5 months after Achilles tendon suture repair, fluid leakage and foreign body reaction occurred at the incision site. He came to our institution for treatment in 2016. We performed the same arthroscopic surgery on him as in the previous case (Fig. 3). A strip was not used, but sterile dressing and cotton compression dressing were applied to the legs. After the surgery, the affected limb was fixed with plaster of Paris to immobilize it. Because wound infection could not be ruled out, intravenous antibiotics were administered, just once after the operation. The postoperative condition of this patient was good.

However, in 2018, the patient noticed a subcutaneous bulge in the Achilles tendon area, localized skin redness, and tenderness. He came to our department for treatment 1 month later. We considered these symptoms to be caused by a foreign body reaction due to a piece of suture that was missed in the previous surgery. Therefore, under local anesthesia, we performed a 1‐cm incision, found the thread, and removed it. No tourniquet was used in this operation, and no postoperative antibiotics were given.

MRI results showed a significant improvement in the Achilles tendon area during follow up compared with preoperatively. Pain at the Achilles tendon was assessed using visual analogue scale (VAS) scores, with 0 indicating no pain at all and 10 indicating the maximum pain. The function and movement of the Achilles tendon were assessed using scores on the American Orthopedic Foot and Ankle Society (AOFAS) Ankle‐Hindfoot Scale^9^ and the Victorian Institute of Sport Assessment Achilles (VISA‐A)^10^ questionnaire as well as the Achilles tendon total rupture score (ATRS)^11^. Because no Achilles tendon sports ability scores were available, we used the Tegner knee motion score^12^ to evaluate the overall lower limb function and recovery of motor ability in patients after surgery. At the last the follow up in 2019 (3 years after the operation), the patient's VAS had improved from 6 preoperatively to 0, his AOFAS score changed from 45 to 100, his VISA‐A score from 33 to 76, his Tegner knee motion score from 1 to 3, and his ATRS from 19 to 92, which indicated that the function and movement of the patient's affected limb had improved satisfactorily.

### Case 3

The present patient was a 53‐year‐old Chinese man experiencing pain in the Achilles tendon area after Achilles tendon suture repair, with the incision was not having healed well after 2 months. The patient came to our institution to seek medical advice in 2019, and we performed endoscopic surgery on him. Poor blood circulation was noted in the operation area, so prophylactic antibiotics were used to prevent infection (only once), and the affected limb was fixed with plaster after the surgery.

However, the Achilles tendon was injured again 2 months after the operation, and he reported pain due to the weight borne by the affected limb because of the fall, but the skin over the Achilles tendon was intact and undamaged, indicating a successful operation. At the last the follow up in 2020 (8 months after the operation), the patient's AOFAS score had changed from 87 preoperatively to 98 postoperatively, his VISA‐A score from 55 to 71, his Tegner knee motion score from 1 to 3, and his ATRS from 25 to 38, which indicated that the function and movement of the patient's affected limb had recovered well.

## Discussion

### Causes of Delayed Wound Healing After Achilles Tendon Suture Repair

The anatomic characteristics of the Achilles tendon include a thin soft tissue envelope and relatively little blood supply^13^, ^14^, making repair of defects difficult and increasing the risk of postoperative infection and delayed wound healing compared with other tissues. In addition, due to the lack of sebaceous glands on the soles and the presence of abundant sweat glands, the foot, especially when wearing airtight shoes, provides a unique environment for the growth of many bacteria, resulting in bacterial breeding^14^, ^15^. Patient factors that affect the occurrence of postoperative complications include age, obesity^16^, ^17^, and frequent tobacco use^18^. Older patients may, therefore, benefit from the use of absorbable sutures during surgery and postoperative antibiotics for a few days after surgery. Wang *et al*.^19^ indicated that steroid use increases the risk of wound healing complications. Dombrowski *et al*.^16^ reported that patients with medical comorbidities, such as diabetes and arrhythmias, at the time of surgery had a significantly increased incidence of postoperative infections (18.0% compared with 6.0% in those without comorbidities).

Several intraoperative factors can contribute to delayed wound healing. Smith *et al*.^20^ demonstrated that prolonged use of tourniquets during foot and ankle surgery can cause hypoxia and inflammation, impeding postoperative wound healing. However, we use tourniquets in approximately 600 open Achilles tendon repair operations every year, for an average time of 60 min, but the incidence of delayed wound healing is very low. Notably, Svedman *et al*.^21^ found that a longer operation time can enhance the production of healing metabolites, potentiate repair processes, and improve patient prognosis. However, a longer operation time can also increase the rate of wound infection after an Achilles tendon operation. Nonabsorbable sutures are generally used in Achilles tendon repair because of their durability and longevity, but they may also cause a local immune response and inflammation^22^, which are critical causes of delayed healing of the Achilles tendon. In addition, a subsequent postoperative wound injury, such as from a fall, also delays wound healing^23^.

In our study, all three patients had local inflammation due to foreign body reactions (nonabsorbable sutures), including one patient with significant local skin necrosis, which delayed wound healing.

### Delayed Wound Healing and Infection After Achilles Tendon Suture Repair

Delayed wound healing and bacterial infection (which further delays wound healing) are common postoperative complications after Achilles tendon suture repair ^24^. To determine whether patients have a deep infection, bacterial culture of secretions is performed. In the case of a deep infection, more tissues require cleaning and transplant may be necessary^25^, which is often complex to perform and is associated with poor postoperative recovery.

In our study, the wounds of the three patients had no purulent discharge. We performed a preoperative wound bacterial culture on each of them and the results were negative. After the operation, all three patients recovered and no infection appeared. Although we have not used arthroscopic surgery to deal with patients with wound infection, we believe that it is suitable for them similarly.

### Conventional Treatment Methods for Delayed Wound Healing after Achilles Tendon Suture Repair

Nonsurgical management is often used in delayed wound healing in patients without inflammation or deep infection. Ankle fixation at 20° plantar flexion can maximize the wound healing potential by maximizing oxygen perfusion to the skin near the Achilles tendon^13^. Simple delayed wound healing can be controlled through the use of wet‐to‐dry dressing or sulfadiazine silver cream^26^, whereas wound healing in large skin defects can be accelerated through negative pressure wound treatment^27^.

However, some types of delayed wound healing cannot be treated conservatively, such as those caused by bacterial infections or foreign body reactions (such as unabsorbable sutures)^22^, ^28^. Deep infection is conventionally treated through thorough wound debridement followed by functional treatment to promote healing of the Achilles tendon's fibrous scar. The wound is kept open for routine irrigation and dressing^29^ until the tissue defect is repaired several days later; the wound can then be closed with sutures. In the case of a foreign body reaction, the debridement procedure should involve removal of all nonabsorbable sutures. In recent years, negative pressure wound therapy has been used after wound debridement of the Achilles tendon site^27^, ^30^, which can speed up wound healing (Table 1).

**TABLE 1:** Treatment of abnormal wound healing after Achilles tendon surgery reported in the literature

Authors	Causes	Treatment	Methods
Schipper and Cohen^26^	Non‐infectious delayed wound healing	Conservative treatment	Keep the wound clean Negative pressure wound therapy for a larger wound dehiscence
	Deep infection	Surgical treatment	Surgical irrigation to and debridement with intravenous antibiotics Remove all infected tissue
Mosser *et al*.^27^	Deep infection	Surgical treatment	Open debridement, lavage, and necrectomy of infected tendon parts Vacuum‐assisted closure (VAC) therapy
Kara *et al*.^28^	Suture granuloma	Surgical treatment	Open surgery to remove granuloma
Fourniols *et al*.^29^	Necrotic infection	Surgical treatment	Open radical debridement of the infected tissue and necrotic Achilles tendon Daily irrigation
Saku *et al*.^30^	Deep infection	Surgical treatment	Intravenous antibiotic treatment Open surgery to debride Infected and necrotic parts of the tendon and peritendineum Negative pressure wound therapy Daily tap water shower and povidone‐iodine and sugar ointment

Nevertheless, the conventional surgical approach is open surgery, which produces a larger wound and, thus, involves a longer recovery period. Moreover, it is necessary to keep the wound open postoperatively for daily cleaning, which may lead to complications such as reinfection. Therefore, surgeons must develop better treatment approaches.

### Advantages of Endoscopic Surgery in the Treatment Of Delayed Wound Healing After Achilles Tendon Suture Repair

We pioneered the use of arthroscopy to treat several patients with delayed wound healing after Achilles tendon suture repair. This technique has the advantage of not requiring the wound to be open postoperatively for multiple irrigations. Arthroscopic treatment not only avoids the uncertainty of open surgery but also improves the visibility of the surgical field, which supports quicker and more thorough elimination of contaminated foci. Moreover, making smaller incisions during arthroscopic surgery accelerates wound healing compared with conventional surgery. Generally, the wound is initially healed when the stitches are removed after 2 weeks, but to ensure that the Achilles tendon no longer has problems, we refer to the conservative time of plaster fixation for acute Achilles tendon rupture, and consider 6 weeks as the recovery period. However, in patients with delayed healing caused by foreign body reactions, arthroscopic surgery may not be able to remove all foreign bodies (threads) at one time, as was seen in Case 2. A small possibility exists of the need for a minor local procedure at a later stage. The doctor should explain this to the patient in advance.

Arthroscopic treatment can greatly reduce the difficulty of postoperative care (e.g. no need for additional irrigation) compared with conventional open surgery^29^, thereby reducing the discomfort and financial burden experienced by patients. This procedure also reduces the need for postoperative antibiotics. Patients in Case 2 and Case 3 received postoperative intravenous antibiotics only once. None of the patients presented with any signs of infection during subsequent follow ups, and all exhibited satisfactory wound recovery. This technique enables patients to resume rehabilitation exercises more quickly than the conventional technique, thus benefiting the recovery of Achilles tendon function^31^. Faster wound healing and recovery also improve patients’ quality of life, through patients being able to self‐care sooner, thus making patients more satisfied with the results.
